# The transcriptional coordination of functional gene clusters is dependent on multiple chromatin remodelers in a haploid strain of the budding yeast, *Saccharomyces cerevisiae*

**DOI:** 10.3389/ffunb.2025.1634150

**Published:** 2025-08-15

**Authors:** Abtsam A. Baadani, Gabrielle F. Coon, Christopher Bui, Mary C. Chidester, Reem S. Eldabagh, James T. Arnone

**Affiliations:** ^1^ Department of Biological and Environmental Sciences, Le Moyne College, Syracuse, NY, United States; ^2^ Department of Chemistry, William Paterson University, Wayne, NJ, United States

**Keywords:** functional gene clusters, biosynthetic gene clusters, *Saccharomyces cerevisiae*, chromatin remodeling, position effects, transcriptional co-expression

## Abstract

The organization of functionally related gene families oftentimes exhibits a non-random genomic distribution as gene clusters that are prevalent throughout divergent eukaryotic organisms. The molecular and cellular functions of the gene families where clustering has been identified vary, and include those involved in basic metabolism, secondary metabolite biosynthesis, and large gene families (e.g. ribosomal proteins). Many of these gene families exhibit transcriptional coregulation, however the roles that clustering plays and the mechanism(s) underlying co-expression are currently understudied. A comprehensive characterization of these relationships would allow for a greater understanding of the implications of genetic editing and engineering to minimize undesired consequences. Here we report the impact of gene clustering and genomic positioning on the expression of large, coregulated gene families in a haploid strain of the budding yeast, *Saccharomyces cerevisiae*. Computational analysis identifies a significant and complex role for chromatin remodeling as a mechanism underlying cluster transcription. Functional dissection of the ‘vitamin metabolic process’, ‘ribosome biogenesis’, and ‘ribosomal protein’ gene families, characterized the roles for *SNF2*, *JHD2*, *HIR2*, *EAF3*, and *yKU70* dependent chromatin remodeling during steady state transcription as well as the transcriptional response to glucose replenishment. Finally, mining and analysis of transcription profiles reveals significant transcriptional differences between the clustered and unclustered subsets within coregulated families under specific stressors.

## 1 Introduction

Decades of characterization and study of the many mechanisms that balance transcription have painted a complex picture of multiple layers that collaborate to properly modulate gene expression on a cellular level across all domains of life (Snel, 2004; Payankaulam et al., 2010; Lelli et al., 2012; Bylino et al., 2020). Broadly speaking, the complexity of regulation scales with the complexity of the organism – with eukaryotic mechanisms evolving greater components than their prokaryotic brethren. At the level of transcription of an mRNA species, this complexity has evolved and extends to include *cis* regulatory DNA sequences, *trans* protein binding factors, and genome management via changes to the underlying chromatin collaborating together (Yáñez-Cuna et al., 2013; Tyagi et al., 2016; Lorch and Kornberg, 2017; Rosanova et al., 2017; Morrison and Thakur, 2021; Dsilva and Galande, 2024; Veitia, 2025). An additional layer contributing to proper gene expression and coordination of the co-expression of gene families that contain multiple components is through their distribution and organization throughout the genome along the chromosome into functional clusters (Rocha, 2008; Nützmann et al., 2018).

The genomic organization of fungi – and eukaryotes overall – lacks the classical operon structure that has been long recognized as a defining characteristic for functionally related genes seen in prokaryotes (Jacob and Monod, 1961). There are, however, incidences of clustered co-expressed, functionally related genes that have been identified in many eukaryotic organisms. Included among these functional clusters include gene families whose protein products include the synthesis of primary metabolites, secondary metabolites, and stoichiometrically balanced proteins (Michalak, 2008; Arnone et al., 2012; Arnone, 2020; Cittadino et al., 2023). Potentially the best characterized among these include the many different biosynthetic gene clusters (BGCs), which are found extensively throughout fungi and oftentimes are of interest due to the bioactivity of the metabolites that they produce (Keller, 2015; Robey et al., 2021). Proper expression of BGCs frequently employ many of the aforementioned layers of gene regulation, including chromatin remodeling (Bok et al., 2009). Understanding this is vital as it allows metabolic engineering to optimize production of many of these products (including alkaloids, non-ribosomal peptides, terpenoids, and polyketides) for commercial, pharmaceutical, and biotechnological applications (Li et al., 2016; Mózsik et al., 2022). Functionally related gene clusters also frequently exist in the context of larger gene networks that are linked via common *cis* regulatory sequences and shared *trans* regulatory proteins that balance transcription of the entire family – which are comprised of both clustered and unclustered, singleton family members found in isolation (Kwon et al., 2021).

The formation of functionally related gene clusters has long been observed as an organizational feature of the proteins that are for the production and biosynthesis of the ribosome in the budding yeast, *Saccharomyces cerevisiae* (Wade et al., 2006). These clusters are necessary for proper transcription of the paired genes, which have been reported to share regulatory mechanisms and lose proper expression when separated and no longer are paired (Arnone and McAlear, 2011; Arnone et al., 2014). Systematic analysis and characterizations revealed that approximately 25% of functionally related gene families exhibit a non-random genomic distribution into clusters, oftentimes colocalizing into genomic regions that are conducive to spatial position effects across a longer chromosomal distance (Eldabagh et al., 2018; Cera et al., 2019). Comparative analyses revealed that although the conservation of clustering is conserved the identity of the clustered genes within large families is not (e.g. different genes comprise the membership of the individual clusters in different organisms) (Arnone et al., 2012; Asfare et al., 2021).

As increasingly sophisticated tools and analytical methods have been developed, a complex picture of the interconnected nature of transcriptional effects acting at as distance has emerged. On a global scale there is a positive correlation in gene expression between all genic neighbors that decays as a function of distance, which is conserved throughout eukaryotes (Quintero-Cadena and Sternberg, 2016). This is an extension of the role that spatial position effects play on a local level. In budding yeast this understanding of spatial influences on transcription has expanded from gene silencing via the telomere position effect (TPE) to the recognition genes frequently alter transcription of their genic neighbors through secondary and tertiary effects referred to as the ‘*Neighboring gene effect*’ (Gottschling et al., 1990; Ben-Shitrit et al., 2012; Atias et al., 2016). Furthermore, artificial induction of non-reciprocal chromosomal translocations results in expressional differences to the loci found within the flanking regions (Nikitin et al., 2008). The widespread incidence of these observations is consistent with a model whereby the formation of functional clusters for large gene families may initially be a spurious evolutionary event, but once formed they are maintained via selective pressures and constraints.

The mechanisms that underlie the transcriptional correlation between proximal genic neighbors is likely to depend on a complex interaction of multiple factors. Chromatin modification and organization is likely to play a significant role in this process on a global level, as it does in gene silencing via the sirtuin family histone deacetylase proteins that is characteristic of the TPE (Rusche et al., 2003). The establishment and maintenance of constitutive heterochromatin is limited to a small number of loci in *S. cerevisiae*, including the telomeres and the cryptic mating loci (Bi, 2014). Many regions of the genome undergo changes to the underlying chromatin during changes in gene expression, including covalent modification to histone tails, changes in histone octamer composition by variants, as well as the localization and positioning of histones at a locus (Weiner et al., 2015; Nocetti and Whitehouse, 2016; Reyes et al., 2021; Chou et al., 2023). These alterations to the underlying chromatin can alter the rate of transcription in myriad ways – masking or revealing *trans* regulatory factor binding sites, changing the stability and strength of histone-DNA contacts, and potentially transcriptional interference (Hainer et al., 2011; Chia et al., 2017).

The focus of this work is to comprehensively characterize the role of chromatin remodeling as a mechanism underlying the transduction and co-expression of gene clusters as well as to identify the transcriptional behavior of clustered genes compared to the unclustered members within different gene families. We analyzed microarray datasets from 165 genetic deletion mutants in *S. cerevisiae* that disrupted the function of more than 30 chromatin modifying complexes (including those with histone modification enzymes, nucleosome remodeling complexes, and assembly complexes) to identify those with roles in co-expression of gene clusters. Computational analysis identified a broad interplay for chromatin remodeling complexes as necessary to maintain proper transcription of the clustered genes to the rest of their gene family. Functional dissection of three representative gene families, the ‘vitamin metabolic process’ (VMP), ‘rRNA processing and ribosome biosynthesis’ (RiBi), and ‘ribosomal proteins’ (RP) families, each require the activity of multiple remodeling complexes to maintain proper steady state expression and induction in response to cell-cycle activation via glucose replenishment within the BY4741 haploid genetic background. Finally, we show that clustered genes deviate from their non-clustered, singleton counterparts in a stress-specific manner, which sheds insight into the varied pressures that may select for the formation and stability of these clusters.

## 2 Methodologies

### 2.1 Gene families and datasets used for computational analysis

Composition, membership, and functional annotations of the gene families used in this analysis were originally described previously (Eldabagh et al., 2018). This analysis focused solely on the gene families identified that exhibited a significant occurrence of gene clusters (using a cutoff of a p-value ≤ 0.05), which were selected for characterization within this study. From the 140 gene families described (using the G.O. Slim descriptor definitions) this led to the inclusion of 38 gene families herein. Chromatin remodeling data was accessed and downloaded from the Gene Expression Omnibus (accession #GSE25909). These datasets represented whole genome, microarray analysis of 165 different yeast deletion mutants of chromatin remodeling genes, and transcriptional differences were identified by comparison to isogenic, wild-type strains throughout steady state growth conditions. Transcriptional changes triggered by the induction of varying budding yeast stress responses were accessed and downloaded from the **Supplementary Material** and the Gene Expression Omnibus (accession #GSE3406) (Gasch et al., 2000; Tirosh et al., 2006).

Transcriptional responses selected for inclusion in our analysis were selected for diversity in the stressor’s effects, and data was mined from microarray datasets that measured the time-course following stress induction. Datasets analyzed included those induced by: 2.5mM (high dose) menadione (triggers oxidative stress), 1mM (low dose) menadione, 2.5mM Dithiothreitol (DTT) (reducing agent that destabilizes protein structures), hyperosmotic shock (shift to 1M sorbitol), hypoosmotic shock (shift from 1M sorbitol), 1.5 mM diamide, and three heat shock stressors (29°C-33°C, 30°C-37°C, and 25°C-37°C) (Gasch et al., 2000). Additionally, five environmental and nutritional stressors were selected, including a heat shock exposure at 37°C, oxidative stress to 0.3 mM H2O2, DNA damage response from exposure to 0.02% MMS, nitrogen starvation from omitting ammonium sulfate from the growth medium, and carbon source transition from 2% glucose to 3% glycerol (Tirosh et al., 2006).

### 2.2 Computational prediction of chromatin remodeling proteins necessary for transcriptional coregulation of clustered genes

The identification of chromatin remodeling genes that disrupt the co-expression of functional gene clusters relative to the rest of the gene family were calculated using methods previously described (Cera et al., 2019). Briefly, the transcriptional disruption for each of the 165 non-essential chromatin remodeling mutant genes were determined by extraction of their microarray expression profiles and compared to an isogenic, wild-type strain of yeast (Lenstra et al., 2011). This analysis focused on determining the significance of each factor to disrupt the clustered subset within each family compared to the entire family by using a hypergeometric probability density function:


$$P=1-\sum_{k}\frac{\left(\frac{K}{k}\right)\left(\frac{N-K}{n-k}\right)}{\left(\frac{N}{n}\right)}$$

whereby the probability, *P*, is determined from the total number of disrupted genes, *K*, the number of genes in the clustered subset, *k*, from the number of genes within each family, *n*, and the total number of measured genes, *N*, within each deletion mutant. The complete set of calculated p-values is provided as **Supplementary Table 1**. For clarity and visualization as a heatmap, the p-values were transformed as the Euclidean distance (deviation) from 1.0 (e.g. the closer to the value 1.0 the more significant the role of a specific complex).

### 2.3 Yeast strains and media

All yeast strains used within this study are listed on **Table 1** and are derived from the BY4741 haploid genetic background. Strains of yeast were cultured and grown on standard enriched yeast growth media, YPAD (1% yeast extract, 2% peptone, 40mg/L adenine sulfate, and 2% dextrose), and incubated at 30˚C with 185rpm shaking. Steady state levels of RNA were taken after 72 hours growth, when cultures of yeast were saturated and post diauxic shift. Gene expression profiles monitoring the transcriptional changes in response to glucose replenishment were performed following 72 hours growth via supplementation with pre-warmed dextrose to a final concentration of 2% within each culture. Samples were taken before (0 min) and after (8 min) addition of glucose for RNA extraction and subsequent analysis.

**Table 1:** Yeast strains used in this study.

Name:	Complete genotype
WT (BY4741)	*MATa his3Δ1 leu2Δ0 met15Δ0 ura3Δ0*
*eaf3Δ*	*MATa his3Δ1 leu2Δ0 met15Δ0 ura3Δ0 eaf3::KanMX*
*hir2Δ*	*MATa his3Δ1 leu2Δ0 met15Δ0 ura3Δ0 hir2::KanMX*
*jhd2Δ*	*MATa his3Δ1 leu2Δ0 met15Δ0 ura3Δ0 jhd2::KanMX*
*snf2Δ*	*MATa his3Δ1 leu2Δ0 met15Δ0 ura3Δ0 snf2::KanMX*
*yku70Δ*	*MATa his3Δ1 leu2Δ0 met15Δ0 ura3Δ0 yku70::KanMX*

### 2.4 RNA isolation

RNA samples were isolated using the Quick-RNA Fungal/Bacterial Miniprep kit (Zymo Research, CA) following the manufacturer’s protocol with modifications outlined as follows. Between 80-100mg fresh yeast samples were obtained by centrifugation (2 minutes at 10,000g at room temperature), and washed once with ddH_2_O. Cell lysis occurred via bead-beating, using a Genie Disruptor (Scientific Industries, NY) for 20 mins at room temperature. In column DNA digestion was performed, using 15-20U DNase I (Zymo Research, CA) for one hour prior to completion of the RNA isolation procedure. RNA was eluted into 30uL of DNase/RNase-Free Water (Zymo Research, CA), with the quality and concentration determined by nanodrop spectrophotometer. RNA was checked for residual gDNA contamination by end-point PCR, and samples that were clear were subsequently used for further analysis.

### 2.5 Reverse transcription and gene expression analysis

Reverse transcription was performed on 1-2ug for RNA using the ZymoScript RT PreMix Kit (Zymo Research, CA) using the following protocol: RNA was diluted into a final volume of 10uL using DNase/RNase-Free Water and 10uL of the ZymoScript RT PreMix was added to each reaction for a total volume of 20uL. Incubation conditions to produce cDNA were 25°C x 2 mins, 42°C x 10 mins, and then 95°C x 1 min, and samples were then stored at -20°C until further use. Analysis of gene expression was performed by quantitative PCR using Sybr green chemistry and a CFX Opus Real-Time PCR System (Bio-Rad Laboratories, Inc., CA). qPCR reactions were performed in a final volume of 20uL, using the iTaq Universal SYBR Green Supermix (Bio-Rad Laboratories, Inc., CA) with a final concentration of 0.5uM for each of the two primers for the reaction, and 2uL from a 1/100 dilution of cDNA as the reaction template. PCR cycling conditions were: 95°C x 3mins for the initial incubation, followed by 45 cycles of: 95°C x 10s, 55°C x 15s, 60°C x 30s and a melt-curve was obtained at the end (55°C to 95°C at a rate of 0.5°C/5s) to determine specificity of the reactions.

At 2–3 biological replicates (RNA isolation and processing) were analyzed, and each qPCR reaction was performed as triplicate technical replicates (the final analysis represents the average of 6–12 individual reactions). All samples that indicated a single peak in the melt curve (-d(RFU)/dT) were included in our final analysis. The change in gene expression was calculated using the 2^(-ΔΔC(T))^ method as previously described (Livak and Schmittgen, 2001). Graphical depiction of the change in gene expression is presented as the 2-log of the relative gene expression compared to the *ACT1* control as our reference gene. A complete list of PCR primers used in this study are provided in **Table 2**.

**Table 2:** PCR primers used within this study.

Gene	Target	Sequence (5’ - 3’)	Family/Position
*BIO5*	FP	AAACGGCAGTCGTTGAGTCT	Vitamin Metabolic Process - Cluster
	RP	CTTTGCCAGGTTCCACTTGC	
*BIO4*	FP	AAGCCACCTTTTTGGGGTCA	Vitamin Metabolic Process - Cluster
	RP	CTCCACCGATGGCAGTTCAT	
*BIO3*	FP	GATGTCGCGGAACTGCTAGA	Vitamin Metabolic Process - Cluster
	RP	TGCATCCGTGAGCACTCTTT	
*BIO2*	FP	CGAGAACCTACGACGACAGG	Vitamin Metabolic Process - Singleton
	RP	TCTTCGCTTTCACCGAGACC	
*EBP2*	FP	AACGCTACCTTACAGAAACG	*Ribi* - Singleton
	RP	TCCGTTAGGCCTGCCTCTATCGAA	
*MPP10*	FP	CGAGGAGGAGGAGGCTATTTAT	*Ribi* - Cluster
	RP	CTTCCTCATCCGCAAATAAGTC	
*MRX12*	FP	ACCACCATTGACCCATACTCTC	*Ribi* - Cluster
	RP	GACCACTTCCATCAGTTCATCA	
*RPS0A*	FP	GACCAGATGGTGTCCACGTT	Ribosomal Protein - Cluster
	RP	ACAGCCCTTTGACCGAAAGT	
*RSM27*	FP	TGGCAAGCTACTACGGCAAT	Ribosomal Protein - Cluster
	RP	AGCACCTTTACCACGACGTT	
*RPS7A*	FP	GAAATCGACGTTGCTGGTGG	Ribosomal Protein - Singleton
	RP	GCCAAGAAGATGACATGACGG	
*ACT1*	FP	ATCGTTATGTCCGGTGGTACC	Reference
	RP	TGGAAGATGGAGCCAAAGC	

### 2.6 Computational analysis of transcription and data visualization

The whole genome transcriptional datasets were extracted, and Pearson’s correlation coefficient (P.C.C.) was calculated for each of the stress and nutritional responses using the *Data Analysis* tool pack in Excel. Prior to analysis, any incomplete data was excluded from the calculations. Every pair-wise combination was calculated, excluding self-comparisons. Data visualization throughout utilizing graphs and heatmaps were generated in Excel and in R as listed in the figure legends (Wickham, 2016; Gu, 2022).

## 3 Results and discussion

### 3.1 Functionally related gene clusters are sensitive to the activity of multiple chromatin remodeling complexes

To systematically characterize the role of chromatin remodeling on the co-expression of functionally related gene clusters, a computational analysis was performed utilizing the previously published genome-wide microarray datasets for 165 chromatin remodeling mutants in the budding yeast, *Saccharomyces cerevisiae* (Lenstra et al., 2011). Previous work from our research group calculated the probability for a specific chromatin remodeler using a hypergeometric probability density function, which identified a series of regulators for both the rRNA and ribosome biogenesis (RRB or RiBi) and the ribosomal protein (RP) gene families (Arnone et al., 2014). This same approach was utilized to analyze the 38 gene families that exhibit a statistically significant, non-random genomic distribution throughout *S. cerevisiae* (Eldabagh et al., 2018).

**Figure 1:**
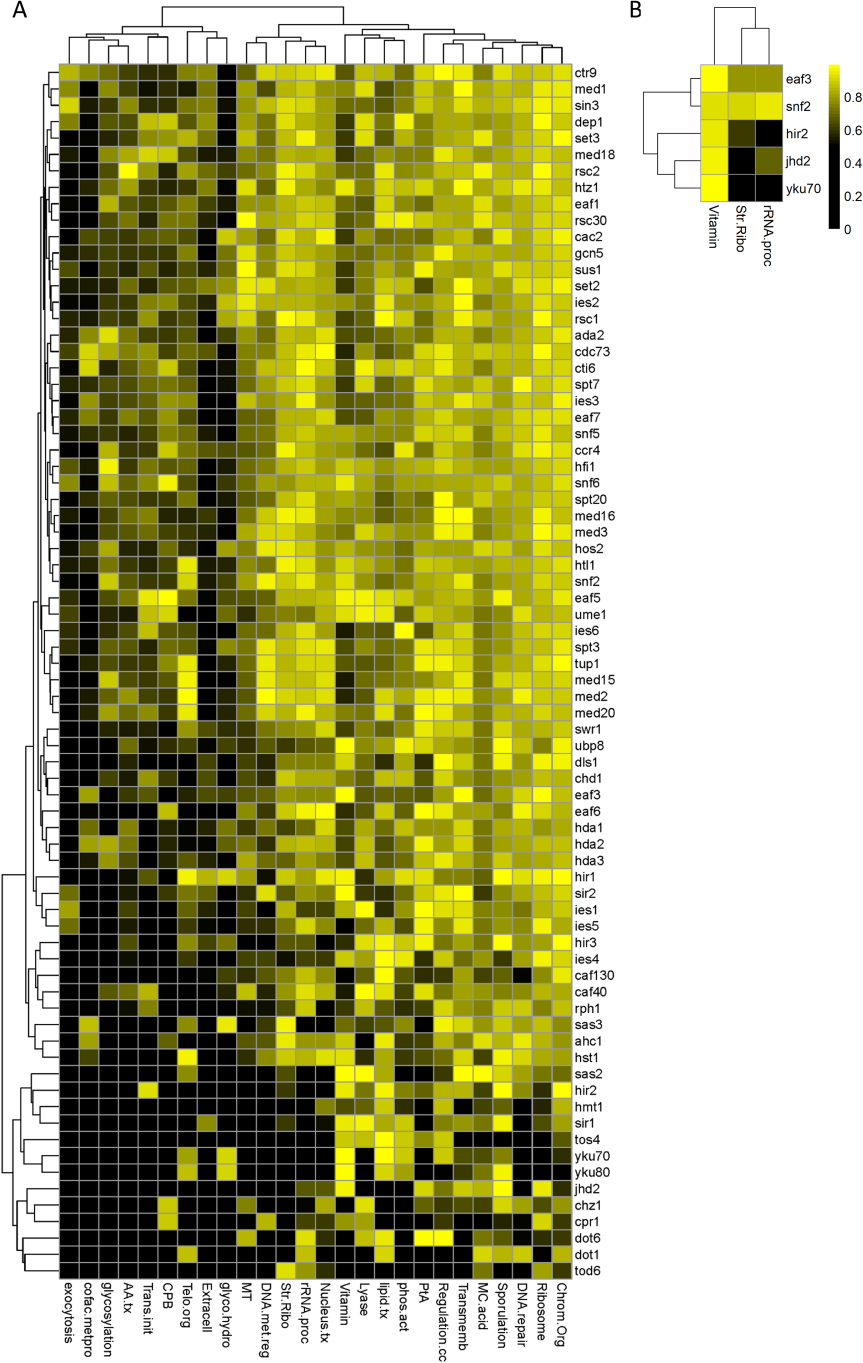
A representative subset of clustered genes within functionally related gene families disrupted by the deletion of many chromatin remodelers. The changes in steady-state transcription were analyzed and the p-value for the significance of the disruption is presented as a heatmap for a representative subset of chromatin remodeler deletion mutants **(A)** and for the subset selected for follow up analysis **(B)**. Data analysis determined the significance of the disruption for the clustered subset of genes within each family relative to the unclustered, singleton members for each deletion mutants relative to wild type gene expression. Hierarchal, k-means clustering organized the families into grouping based on their behavior relative to each remodeling mutant. (cofac.metpro, cofactor metabolic process; AA.tx, Amino acid transport; Trans.init, translational initiation; CPB, cytoskeletal protein binding; Telo.org, telomere organization; glyco.hydro, hydrolase activity, acting on glycosyl bonds; MT, methyltransferase activity; DNA.met.reg, regulation of DNA metabolic process; Str.Ribo, structural constituent of the ribosome; Nucleus.tx, nuclear transport; Vitamin, vitamin metabolic process; lipid.tx, lipid transport; phos.act, phosphatase activity; PtA, peptidase activity; Regulation.cc, regulation of cell cycle; Transmemb, transmembrane transport; and MC.acid, monocarboxylic acid metabolic process).

This comprehensive analysis was completed and the p-values for the significance of each chromatin remodeling mutant to disrupt the clustered subset of genes relative to the entire functionally related gene family was compiled **Supplementary Table 1**). To visualize this wealth of data and look for patterns, the data was transformed to the Euclidean (linear) distance and subsequently converted into a heatmap (**Figure 1A**; **Supplementary Figure 1**). The analysis was allowed to cluster (k-means clustering) and there were three broadly defined groupings for the roles of the many different chromatin remodelers analyzed: there are chromatin remodeling mutants that disrupted transcription globally, mutants that disrupted transcription within a small subset of families, and mutants that seemed to have a negligible effect based on our analysis (**Supplementary Figure 1**).

This approach was successfully able to identify some of the previously identified regulon specific factors, including those of Dot6p, Tod6p, and Sin3p (via an interaction mediated by the sin-three binding protein, Stb3p) that integrate signal transduction pathways to the RRB family, as well as the global role of that Sin3p plays as a component of the Sin3p-Rpd3p histone deacetylase complex (Silverstein and Ekwall, 2005; Liko et al., 2007; Lippman and Broach, 2009; Zhu et al., 2009). Global roles were also observed for Snf2p (a catalytic subunit of the highly conserved SWI/SNF complex), Med20p (a component of the Mediator complex), and many others (Kornberg, 2005; Smith and Peterson, 2005). Looking at this analysis from a broader perspective, the major takeaway is that there is a complex role for chromatin remodeling in the co-expression of functional gene clusters. The sheer magnitude of disruption seen for many of the chromatin remodeling mutant strains – approximately 75% exhibit severe disruption in multiple mutant backgrounds – is likely due to a combination of direct and indirect, epistatic effects. The most parsimonious model that we can postulate is that there are many different mechanisms that can disrupt the co-existence of functionally related gene clusters, making them rather sensitive to this layer of eukaryotic gene regulation.

### 3.2 Validation of the role of chromatin remodelers on the steady state transcription

Following the identification of chromatin remodelers that are necessary for functionally related co-expression of BGCs above, a subset was selected for empirical verification, validation, and further analysis. Five chromatin remodeling mutants were selected, choosing a representative subset that exhibit a diverse series of molecular functions. This analysis focused on: *EAF3* (a component of both the Rpd3p HDAC and NuA4 HAT complexes), *HIR1* (a component of the HIR nucleosome assembly complex), *JHD2* (a JmjC domain family histone demethylase that functions to promote global demethylation of H3K4), *SNF2* (the catalytic subunit of the SWI/SNF chromatin remodeling complex), and *yKU70* (required for the formation of telomeric heterochromatin) (Ashburner et al., 2000; The Gene Ontology Consortium et al., 2023). The choice of gene families selected for analysis consisted of the ribosomal protein (RP), which are annotated with the ‘*structural constituent of the ribosome’*, the ‘*rRNA processing*’ (RiBi), and the ‘*vitamin metabolic processing*’ (VMP) gene families. The choice of the RP and RiBi families were made as these are both extensively studied, characterized, and conserved (Arnone et al., 2012). The selection of the VMP gene family was made to complement the RP and RiBi families, as the VMP as a family is less well studied – however their genomic distribution is the most significant (lowest p-value, null hypothesis is obtaining the exact clustering relationship observed by chance) in previous analysis (Eldabagh et al., 2018).

**Figure 2:**
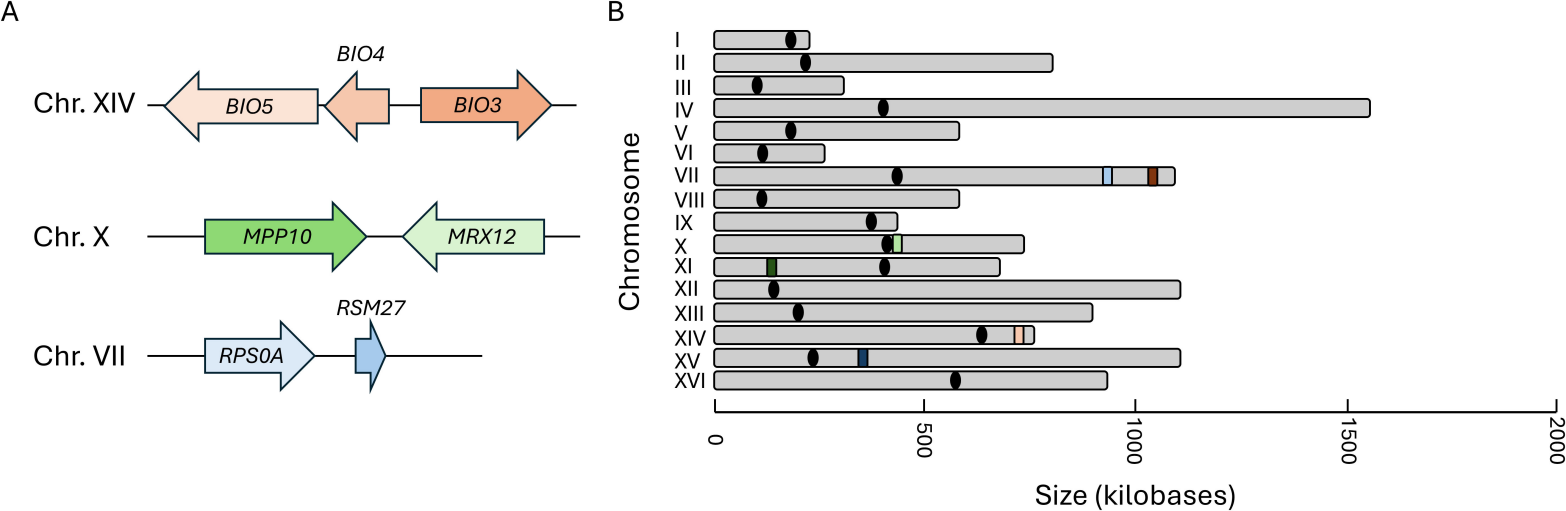
The genomic conformation and distribution of genes selected for expression analysis. The organization of the genetic locus of the three representative sets of clustered genes **(A)**, and the relative genomic distribution of each cluster and the singleton member of each set is depicted in **(B)**. The locus identifiers are depicted as peach/orange for the vitamin metabolic process genes, green for the Ribi genes, and periwinkle/blue for the RP genes. The lighter shaded represents the position of the clustered locus and the darker color represents the position of the singleton for each family.

Representative clustered genes within the RiBi, RP, and VMP gene families were selected for gene expression analysis along with one singleton member (to serve as a control) from each set. The orientation of the clusters and the genomic distribution of all loci tested were mapped (**Figure 2**). The specific cellular and molecular functions of each gene selected for this analysis have been compiled and can be found in the **Supplementary Materials** (**Supplementary Table 2**). The VMP cluster chosen for analysis is the triplet *BIO5-BIO4-BIO3*, found on chromosome 14, which is compared to the singleton *BIO2* located on chromosome 7. The RiBi gene cluster chosen for study is the *MPP10-MRX12* gene pair that is localized in a convergent orientation on chromosome 10, proximal to the centromeric region on the short arm of the chromosome. For comparison, *EBP2*, which is found on chromosome 11, was selected as the singleton member of this gene set. The RP gene cluster chosen is *RPS0A-RSM27*, a tandem gene pair that is found on chromosome 7. The singleton member of the RP gene family is the *RPS7A* gene found on chromosome XV; all three of the RP genes selected were chosen as being components of the same ribosomal structure (they are all components of the small ribosomal subunit). The positions of nucleosomes were extracted to identify their occupancy within the promoter regions for each gene cluster and the singleton controls that were selected for follow up analysis (Jiang and Pugh, 2009). Consistent with previous analyses, it was found that all three of the RP genes have a nucleosome free region (NFR) within their promoter regions and all three of the Ribi genes also contain an NFR upstream of the site of transcription initiation (**Supplementary Table 3**) (Rossi et al., 2021). The VMP triplet contains a single NFR within the promoter region of the gene that is located within the middle of the cluster, *BIO4*, the two flanking genes and the singleton, *BIO2* do not contain an NFR region.

**Figure 3:**
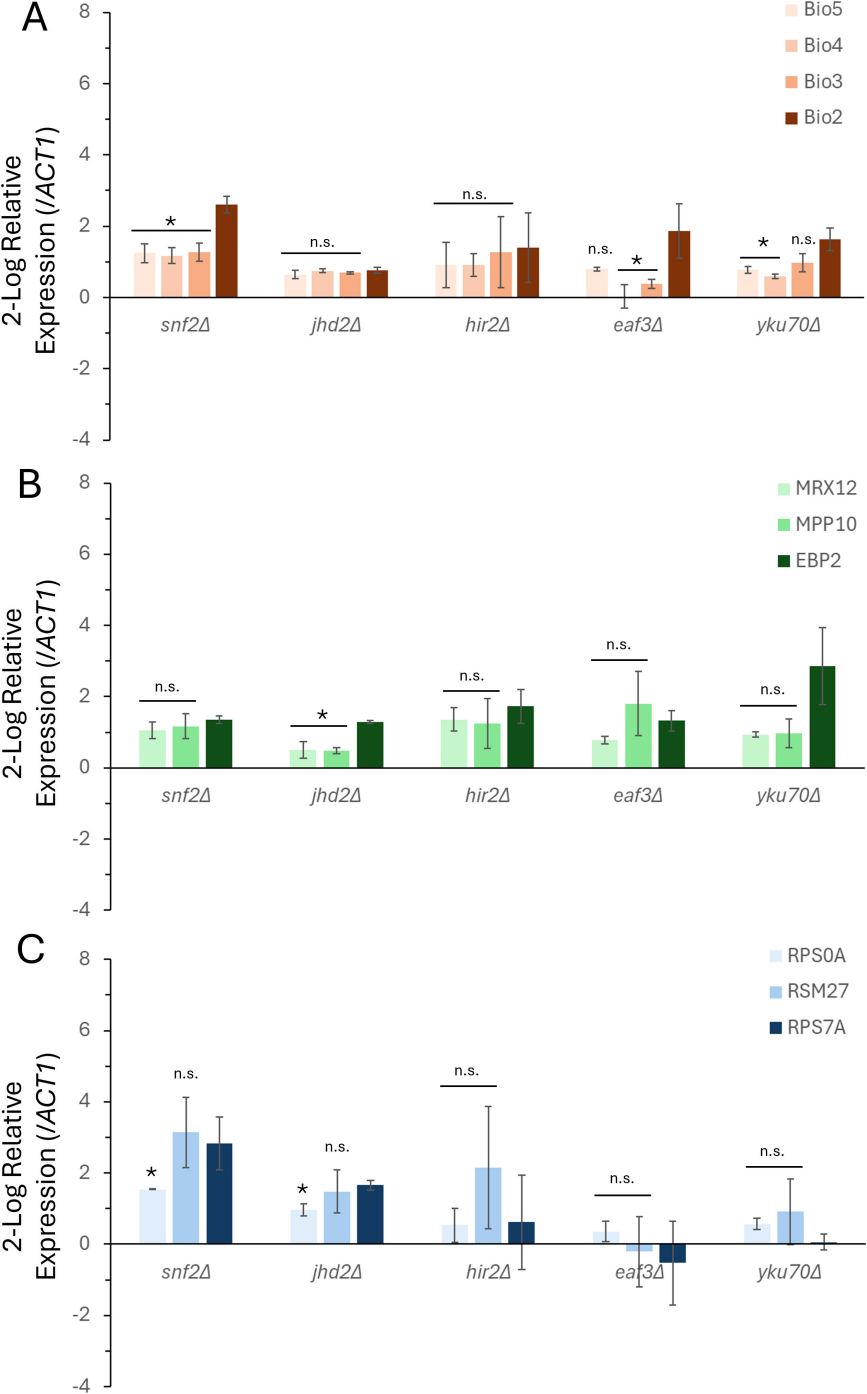
The steady-state levels of transcription for functional gene clusters following the diauxic shift is dependent on multiple chromatin remodeling proteins. Gene expression profiles were measured and quantified for the steady state level of transcription following 72-hours of growth for the VMP **(A)**, RiBi **(B)**, and RP **(C)** gene families. The relative levels of gene expression were calculated compared to the wild type strain of yeast, and the expression of *ACT1* (actin) was the reference gene. Gene expression was calculated using the 2^(-ΔΔC(T))^ method, and the levels of expression are presented as the 2-log fold change for each gene. The data represents the average of two independent biological replicates and triplicate technical replicates (+/- the S.E.M.). *indicates significance (p < 0.05), students t-test.

The VMP, Ribi, and RP data for each chromatin remodeler selected for follow up analysis was extracted for ease of comparison (**Figure 1B**). These data analyses were obtained during steady state (logarithmic) growth conditions, and there are both similarities differences in the coordination of clusters relative to the rest of each gene family. Our approach focused on extending the understanding of this mechanism mediating transcriptional co-regulation, and gene expression measurements were obtained following the post-diauxic shift in metabolism. The VMP gene cluster analyzed was completely disrupted within the *snf2Δ* mutant and exhibited partial disruption in both the *eaf3Δ* and the *yku70Δ* genetic backgrounds (**Figure 3A**). There was no significant difference measured within either the *jhd2Δ* or the *hir2Δ* backgrounds. The RiBi gene cluster was disrupted in the *jhd2Δ* background only, there was no statistically significant transcriptional difference measured within any of the other genetic backgrounds (**Figure 3B**). The RP gene cluster was disrupted in both *snf2Δ* and the *jhd2Δ* mutants; in both cases the disruption represented a statistically significant difference at this locus, uncoupling the pair from each other (**Figure 3C**). There was no measurable difference in expression observed within the *hir2Δ*, *eaf3Δ* or the *yku70Δ* strains.

### 3.3 The transcriptional response to glucose replenishment is dependent on many chromatin remodeling complex components

**Figure 4:**
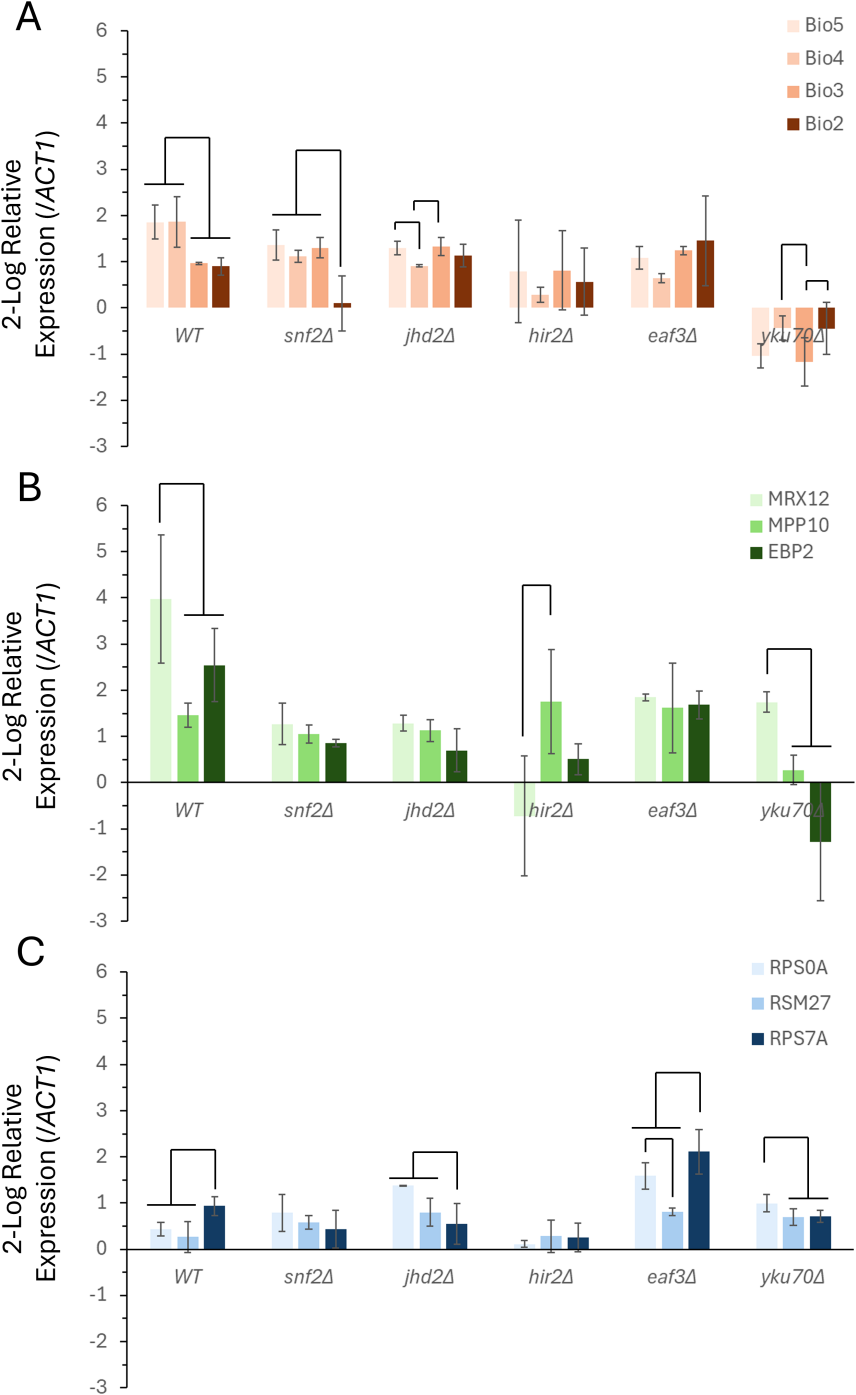
The transcriptional response of functional gene clusters to glucose repletion is mediated by multiple chromatin remodeling proteins. Gene expression profiles were measured and quantified to determine the transcriptional response follow glucose replenishment for the VMP **(A)**, RiBi **(B)**, and RP **(C)** gene families. The relative levels of gene expression were calculated comparing levels of expression prior to and following the addition of 2% glucose to post-diauxic shift cells, and using the expression of *ACT1* (actin) as the reference gene. Gene expression was calculated using the 2^(-ΔΔC(T))^ method, and the levels of expression are presented as the 2-log fold change for each gene. The data represents the average of two independent biological replicates and triplicate technical replicates (+/- the S.E.M.). The connector lines indicate significant differences in levels of expression (p < 0.05), ANOVA test of variance.

In addition to chromatin remodelers mediating the absolute levels of expression of these gene clusters, they may also be necessary to mediate the transcriptional response to a changing extracellular environment. To identify this role, the same three gene clusters were monitored in each of the five-chromatin remodeling mutant genetic backgrounds during glucose repletion to activate the cell cycle. Post-diauxic shift cultures were spiked with dextrose to a final concentration of 2%, and gene expression differences were determined following eight minutes of induction (**Figure 4**). The changes in transcription were determined for the wild-type strain of yeast and each of the five mutant strains in this analysis.

The VMP triplet cluster and the control, *BIO2*, are all induced following glucose replenishment, with *BIO5* and *BIO4* expressed at a significantly higher level than *BIO3* and *BIO2* (**Figure 4A**). In each of the five chromatin remodeler mutants the co-expression of this gene cluster is disrupted. *HIR2* and *EAF3* are both required for the full and complete induction of both *BIO5* and *BIO4*. Within the *snf2Δ*, *jhd2Δ*, and *yku70Δ* backgrounds there is a more complex relationship, with a reduction in the induction of specific components observed. The *snf2Δ* strain has a reduction in the *BIO5*, *BIO4*, and the singleton *BIO2* transcripts, however the *BIO3* transcript was largely unchanged in this background. The changes seen in the *jhd2Δ* strain show a failure to induce *BIO5* and *BIO4* compared to the wild-type strain, with *BIO4* specifically deviating to the greatest degree – showing a statistically significant reduction relative to the other members within this gene cluster. The *yku70Δ* background was particularly disruptive to the VMP as a whole – with an overall downregulation in seen in the entire family.

The RiBi gene family results in a strong upregulation in response to the addition of glucose to the post-diauxic cultures (**Figure 4B**). The clustered genes, *MPP10* and *MRX12*, as well as the singleton, *EBP2*, were all strongly induced at the time point that was measured in the wild-type background – with *MRX12* showing the most statistically significant upregulation. This specific upregulation of *MRX12* is lost in both the *snf2Δ* and the *jhd2Δ* strains. In each case the overall induction of this family was also reduced in both backgrounds. The upregulation of *MRX12* was also lost in the *eaf3Δ* strain, however the overall change in the expression of both *MPP10* and *EBP2* was largely unaffected by the loss of this chromatin remodeler. The transcriptional differences measured in *hir2Δ* disrupted *MRX12*, which failed to induce with *MPP10* and *EBP2*. As was the case with the VMP gene family, the *yku70Δ* strain exhibited the most severe transcriptional differences compared to the wild-type strain, with both *MPP10* and *EBP2* failing to induce, although *MRX12* maintained a degree of induction that deviated in a statistically significant manner compared to the other two RiBi genes.

The RP genes also were induced in response to the pulse of glucose; however, their overall level of induction was more modest than both the VMP and RiBi gene families (**Figure 4C**). In the wild-type background *RPS7A* was induced to the greatest degree, and it deviated in a statistically significant manner from the *RPS0A* and *RSM27* cluster. The transcriptional induction of this cluster is disrupted in the *snf2Δ*, *jhd2Δ*, and the *hir2Δ* strains – their induction of both *RPS0A* and *RSM27* was higher relative to the *RPS7A* control in each of these genetic backgrounds. In the *eaf3Δ* and the *yku70Δ* background *RPS0A* was effectively uncoupled from its neighbor in the cluster, *RSM27*.

### 3.4 Transcriptional correlation of the vitamin metabolic process gene family differs for the clustered versus singleton subset

**Figure 5:**
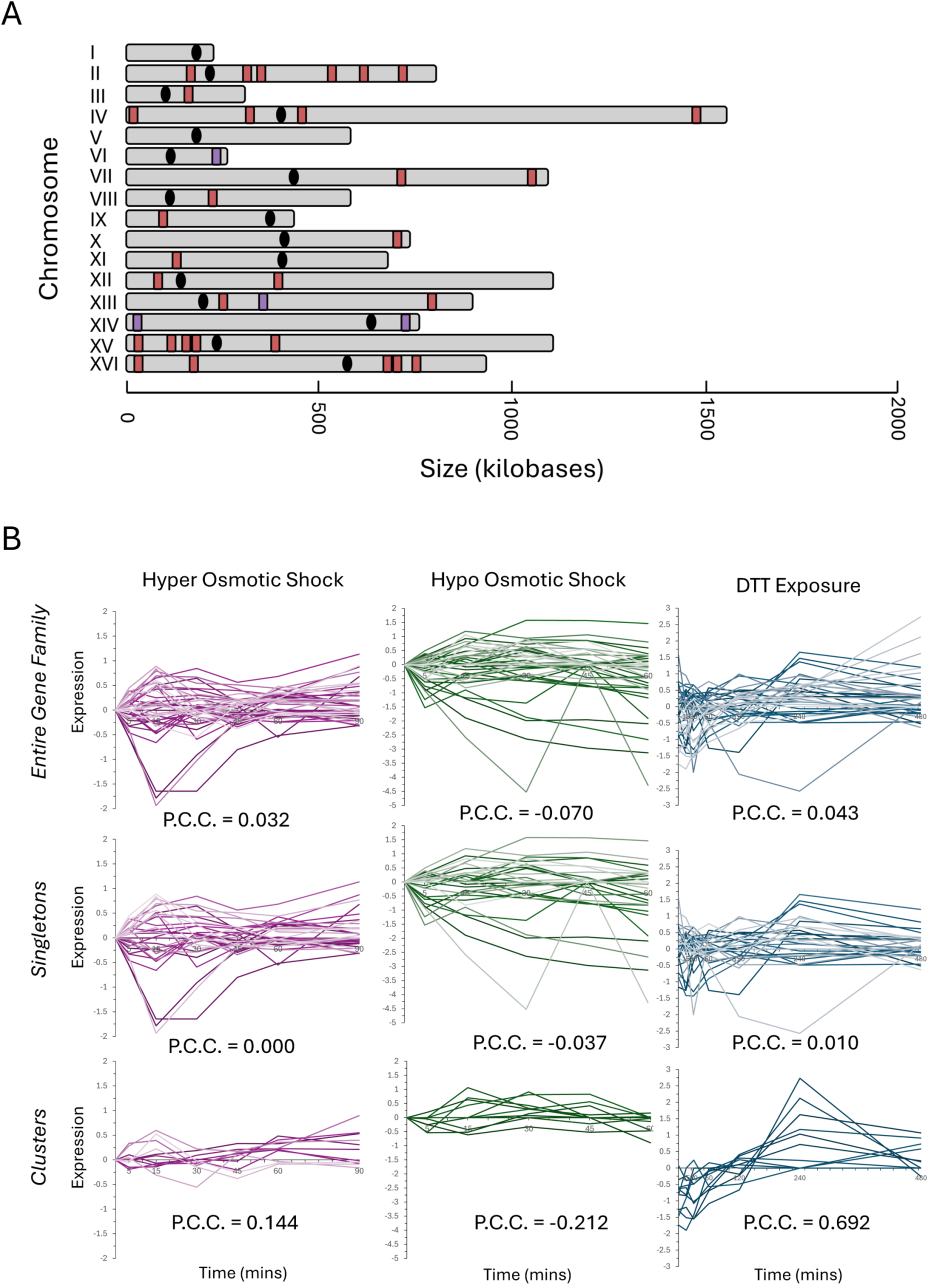
The genomic distribution and transcription profiles of the ‘vitamin metabolic process’ gene family. The 43-member gene family annotated with a molecular function involving VMP gene family (GO:0006766) was mapped, and the genomic distribution is depicted **(A)**. The red markers indicate singleton members of the family, and the purple markers indicate a locus where VMP clustered genes are present. The transcription profiles for the VMP genes in response to three representative stress responses are depicted in **(B)**. The entire 43 gene family is plotted on the top row, the singleton members are plotted in the middle row, and the clusters are plotted on the bottom row. Pearson’s correlation coefficient was calculated and is present on each graph.

The VMP, RiBi, and RP families demonstrate a difference in the transcriptional similarity for the clustered subset compared to the singleton subset throughout the cell cycle (Eldabagh et al., 2018). As the RiBi and RP gene families have both been previously characterized to a much greater extent than the VMP gene family, expanded our analysis to focus on this set. The genomic distribution of the VMP gene family was mapped to each chromosome (**Figure 5A**), and, aside from the highly significant incidence of gene clusters, there was no other significant feature that defined the VMP family’s genomic distribution.

One potential selective pressure that could be driving the formation and maintenance of functionally related gene clusters is that clustered genes may exhibit a greater transcriptional coherence (correlation in transcription), which could be parsed by computational analysis of the transcriptional response to different stressors. To test this possibility, the transcriptional response time-course dataset measuring the expressional differences for the VMP was extracted for three distinct stressors: hyper osmotic shock, hypo osmotic shock, and the response to DTT (a strong reducing agent that disrupts cellular proteostasis) (Tirosh et al., 2006). The expression of the entire VMP family was plotted versus time (**Figure 5B**, top row), and then separate plots for both the singleton family members and the clusters were separated for clarity (**Figure 5B**, middle row and bottom row, respectively). The transcriptional similarity of the family and each subset was calculated by determination of the pairwise Pearson’s correlation coefficient (PCC). The behavior of the entire VMP family was weak under each of these three stress conditions, there is a weak correlation during induction of a hyper osmotic shock (PCC = 0.032) and in response to DTT (PCC = 0.043) and exhibiting a weak anti-correlation during the hypo osmotic shock (PCC = -0.070).

Parsing the data and reanalyzing the transcriptional correlation for the clustered versus the unclustered subset of genes uncovered stark differences in the transcriptional behavior for each set during each stressor. The differences in the PCC for the clustered subset of genes during each stressor was statistically significant and deviated from the singleton subset in all three instances. The difference in the PCC during the response to DTT was the greatest (PCC_singletons_ = 0.010 versus PCC_clusters_ = 0.692), although there were large differences seen for both hyper osmotic shock (PCC_singletons_ = 0.00 versus PCC_clusters_ = 0.144) and for hypo osmotic shock (PCC_singletons_ = -0.037 versus PCC_clusters_ = -0.212). This initial result paints a picture where the clustered genes do not simply behave in a simple, linear fashion to different transcriptional stimuli.

### 3.5 Transcriptional correlation of the clustered subset of functionally related gene families deviates from the unclustered subset under the induction of specific nutritional and stress responses

**Figure 6:**
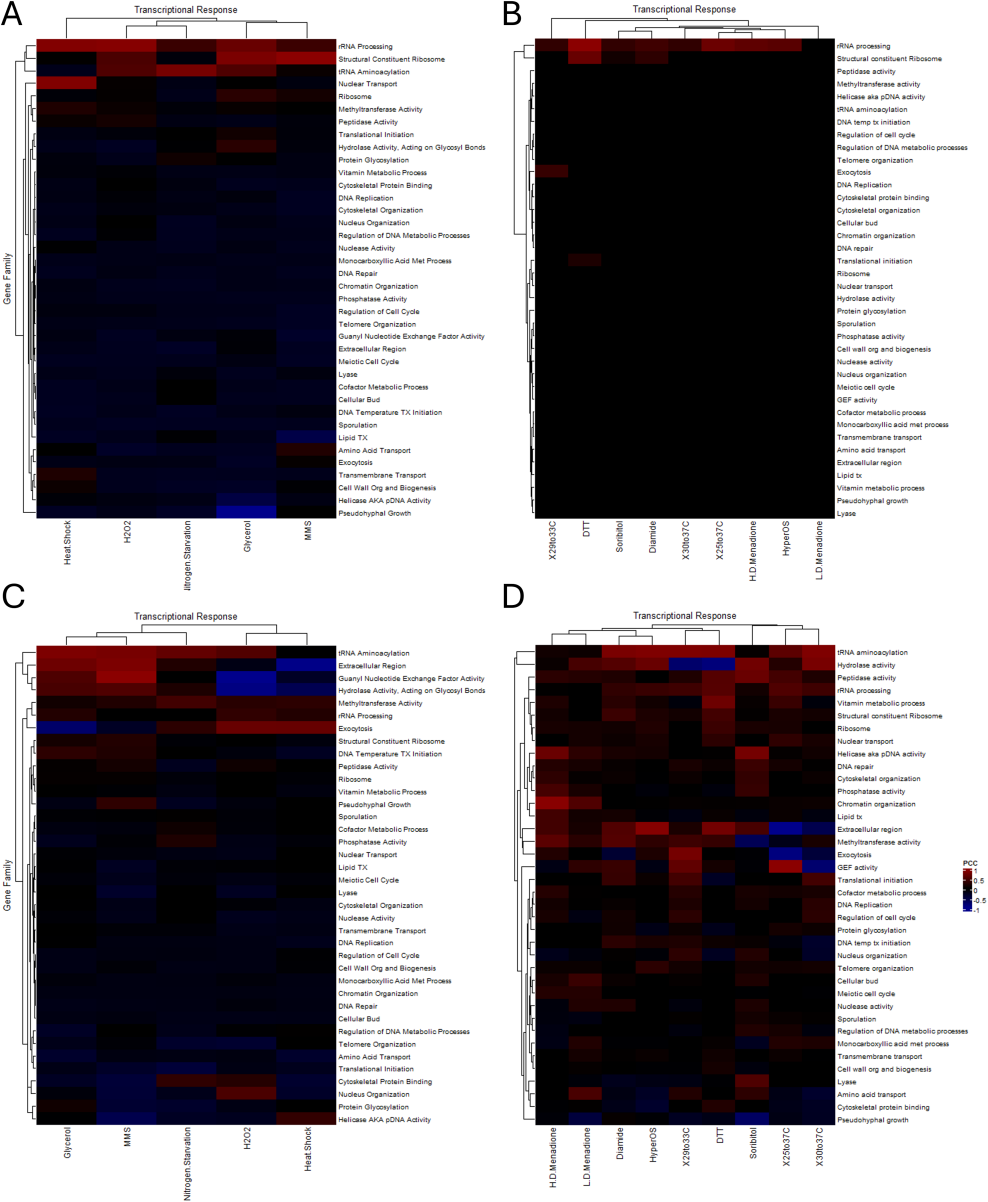
The transcriptional correlation of functionally related gene families to nutritional and stress responses. Heatmaps depicting the P.C.C. for unpaired genes **(A, B)** and clustered genes **(C, D)** from stress and nutritional transcriptional responses from the analysis of the Tirosh et al. **(A, C)** and the Gasch et al. **(B, D)** datasets. Warmer colors indicate positive correlation and cooler colors indicate anticorrelations. Abbreviations for family names is the same as previously listed.

To test if the trend seen in the VMP genes was an outlier or if there are differences in the correlation seen between the clustered versus the singleton members is a feature of gene families, a systematic analysis was performed for each of the 38 gene families where clustering is prevalent. In all, fourteen different stress and nutritional stimuli were selected for analysis (Gasch et al., 2000; Tirosh et al., 2006; Eldabagh et al., 2018). The transcriptional response profiles were extracted for each gene family, they were parsed into the clustered and the unclustered subset, the pairwise PCC was determined, and the transcriptional similarity was visualized by a heatmap (**Figure 6**). The data was sorted by k-means clustering to identify patterns between families and different stressors.

**Figure 7:**
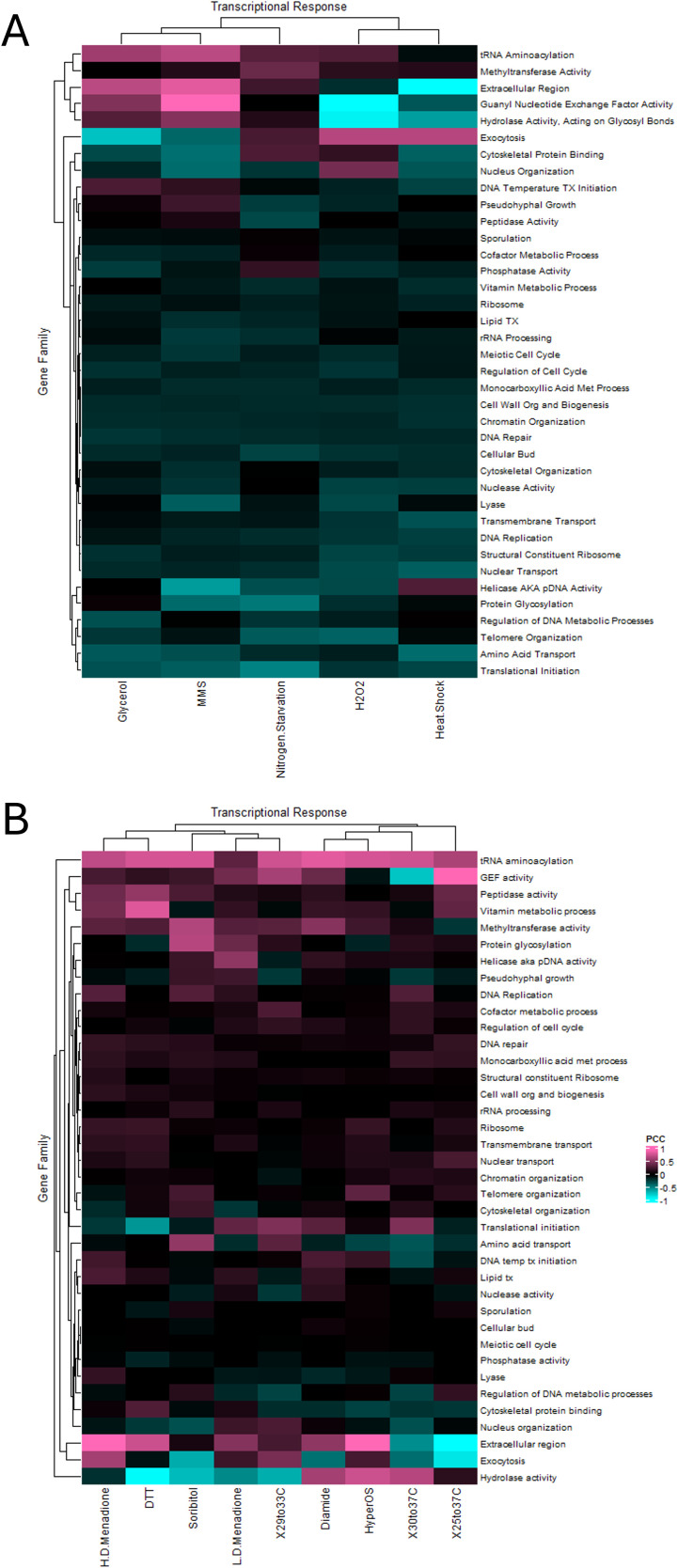
The deviation in transcriptional correlation between the clustered versus the unclustered subset of functionally related gene families during nutritional and stress responses. Heatmaps depicting the deviation (Euclidean distance) in the P.C.C. when comparing the unpaired genes to their clustered gene counterparts from analysis of the Tirosh et al. **(A)** and the Gasch et al. **(B)** datasets. Warmer colors indicate a greater positive difference in correlation and cooler colors indicate a greater negative anti-correlation.

In each of the fourteen different transcriptional responses analyzed there was a striking difference seen when comparing the singleton members of each gene (**Figures 6A, B**) to the clustered members (**Figures 6C, D**). Broadly speaking, the many different families exhibited a broad, albeit weak correlation in their expression that appeared to be magnified in the clustered subset of genes. This appeared to vary based on the mechanism inducing and transducing the response, and in many cases the magnitude of response (represented by the warmth of the colors) reflected that trend. Due to the weak correlation that was seen in many cases when comparing the heatmaps (e.g. comparing **Figures 6A–C** and **Figures 6B–D**) the Euclidean space between each subset was calculated and visualized via heatmap (**Figure 7**). This analysis highlights the difference in transcriptional correlation for the clustered subset of genes clearly, specifically the variance seen across stressors (each row) and between gene families (each column). The emerging picture from these computational analyses is that the transcriptional behavior of the clustered subset of genes represents the complexity in identification of selective pressures that may act to form and stabilize this genomic distribution. This correlation likely adds to the challenges involved in fully characterizing and understanding the impact of the spatial influences and consequences on localized gene expression.

## 4 Conclusions

It has been long recognized that the two-dimensional organization and conformation of genes along the chromosomes has profound implications for their expression based on position effects in model systems via mechanisms such as position effects (Gottschling et al., 1990; Elgin and Reuter, 2013). This effect is broadly conserved throughout evolutionary relationships – up to and including humans, where this effect may be a contributing factor to certain diseases (Ottaviani et al., 2008). The advent of greater resources and technologies in the intervening decades has revealed that position effects on gene expression can be quite significant, and highlights the importance of their impact as both a naturally regulatory mechanism as well as their implications for genetic modification and bioengineering applications (Chen and Zhang, 2016; Wu et al., 2017; Arnone, 2020). Here, we have presented a comprehensive, computational analysis and characterization of the roles that spatial positioning plays in the coregulation of the functional clusters of genes, focusing our analysis on a haploid strain of the budding yeast, *Saccharomyces cerevisiae*. This includes the role of chromatin remodeling complexes in the transcriptional coordination of functionally related, clustered genes during steady-state growth.

Our follow-up verification of this analysis focused on three representative gene families, the VMP, RiBi, and RP families. Our work verified our computational analysis and extended these observations to the role of five representative mutants that are known to alter chromatin (*SNF2*, *JHD2*, *HIR2*, *EAF3*, and *yKU70* null mutants) in transcription in post-diauxic shift levels of expression as well as during the transcriptional response to glucose replenishment. This work revealed a complex relationship between proper chromatin state and co-expression of functional clustered genes in all three families. While it was beyond the scope of this study to parse the direct versus indirect and epistatic roles of each, it is evident that clustered genes are likely subject to both mechanisms. The complexity of this interplay ultimately led us to focus on the behavior of clustered genes compared to their unclustered counterparts. Our initial focus was on the VMP gene family, whereby the clustered versus singletons exhibited marked transcriptional divergence when analyzed for their transcriptional correlation. This was not unique to this gene family, rather this behavior is widespread and is a characteristic of these clusters. The clustering of genes within the same biosynthetic pathway can buffer their expression in a manner that provides a selective advantage and survival, such as the *GAL* metabolic cluster that limits the accumulation of the toxic intermediate, galactose-1-phosphate, that results in cytotoxicity when this triplet is separated (McGary et al., 2013). One caveat to our study is the focus on a haploid strain of yeast, which limits the applicability of the analysis reported herein. A more thorough analysis is needed to extend these results to a diploid strain (and to diploid organisms) and is beyond the scope of this work. One study that has performed such an analysis within a diploid genetic background was focused on the *GAL* gene cluster, finding cell viability differed significantly between haploinsufficient genetic backgrounds when clustering was maintained versus lost (e.g. *cis* versus *trans*) (Xu et al., 2019). Whether this is the norm has yet to be elucidated at this point.

This work expands our current understanding to identify conditions where the clustered subset of genes deviates from the rest of the gene family, which may point to conditions or stressors that ultimately drive the formation and maintenance of functional clusters in budding yeast. These findings are likely not limited to just *Saccharomyces cerevisiae*, as many of the defining characteristics that this work built upon have been found to be widely conserved throughout divergent *Ascomycete* lineages and beyond (Hagee et al., 2020; Cittadino et al., 2023). A thorough understanding of this phenomenon is essential, as it is increasingly apparent that these local spatial effects can have profound implications, and be severe enough to alter the cellular phenotype via secondary and tertiary effects (Ben-Shitrit et al., 2012; Atias et al., 2016; Hartnett et al., 2025). The functional dissection and understanding of this as a regulatory mechanism will undoubtedly lead to safer gene editing design, particularly when performing metabolic engineering – which frequently rely on haploid strains to minimize unnecessary complications (Hong and Nielsen, 2012; Wang et al., 2023).

## Data Availability

The datasets presented in this study can be found in online repositories. The names of the repository/repositories and accession number(s) can be found in the article/**Supplementary Material**.

## Supplementary material

Supplementary Figure 1 The response of the entire set of clustered genes within functionally related gene families following the deletion of 165 chromatin remodeling proteins. The changes in steady-state transcription were analyzed and the p-value for the significance of the disruption is presented as a heatmap for the entire 165 chromatin remodeling mutants across the 38 gene families chosen for inclusion in this analysis. Data analysis determined the significance of the disruption for the clustered subset of genes within each family relative to the unclustered, singleton members for each deletion mutants relative to wild type gene expression. Hierarchal, k-means clustering organized the families into grouping based on their behavior relative to each remodeling mutant. (Abbreviations: cofac.metpro = cofactor metabolic process, AA.tx = Amino acid transport, Trans.init = translational initiation, CPB = cytoskeletal protein binding, Telo.org = telomere organization, glyco.hydro = hydrolase activity, acting on glycosyl bonds, MT = methyltransferase activity, DNA.met.reg = regulation of DNA metabolic process, Str.Ribo = structural constituent of the ribosome, Nucleus.tx = nuclear transport, Vitamin = vitamin metabolic process, lipid.tx = lipid transport, phos.act = phosphatase activity, PtA = peptidase activity, Regulation.cc = regulation of cell cycle, Transmemb = transmembrane transport, and MC.acid = monocarboxylic acid metabolic process).

Supplementary Table 1 Calculated p-values for the significance of all 165 chromatin remodeling mutants across the 38 gene families chosen for inclusion in this analysis.

Supplementary Table 2 Cellular and molecular functions of the genes selected for gene expression analysis.

Supplementary Table 3 Nucleosome free regions flanking analyzed gene set.
