# Supplementary figures and images for: The transcriptional coordination of functional gene clusters is dependent on multiple chromatin remodelers in a haploid strain of the budding yeast, Saccharomyces cerevisiae
Source: Front Fungal Biol. 2025 Aug 15;6:1634150. doi: 10.3389/ffunb.2025.1634150 (PMC12394982; doi:10.3389/ffunb.2025.1634150)

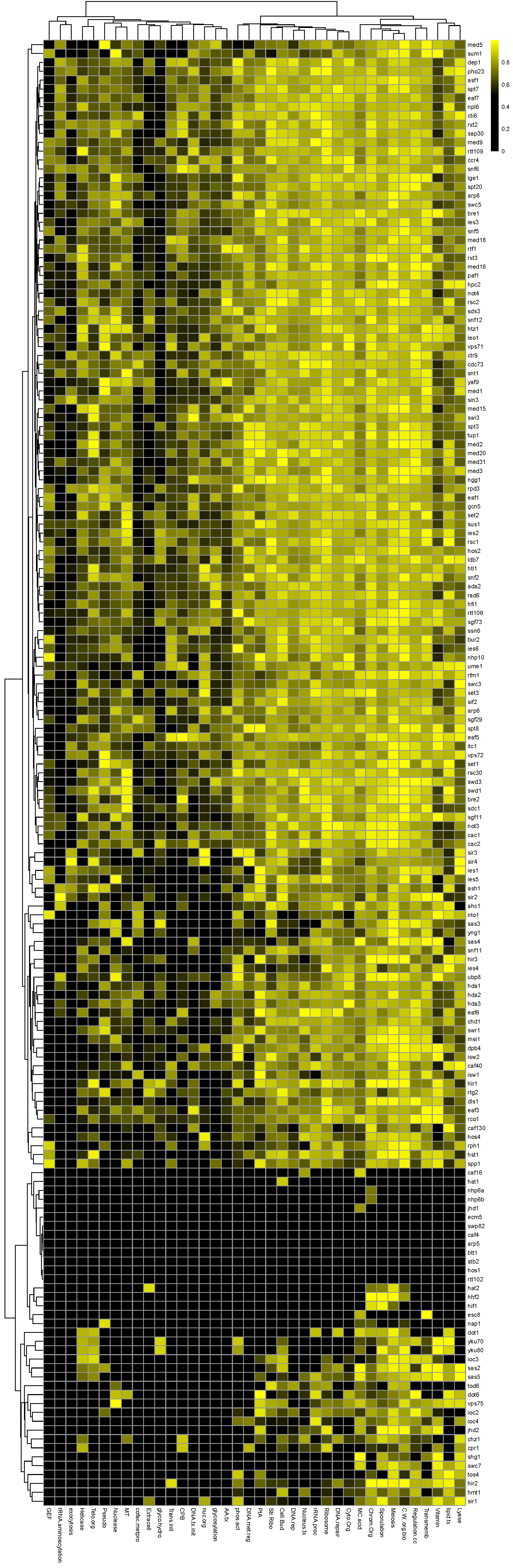

Supplement: Supplementary Figure 1 — The response of the entire set of clustered genes within functionally related gene families following the deletion of 165 chromatin remodeling proteins. The changes in steady-state transcription were analyzed and the p-value for the significance of the disruption is presented as a heatmap for the entire 165 chromatin remodeling mutants across the 38 gene families chosen for inclusion in this analysis. Data analysis determined the significance of the disruption for the clustered subset of genes within each family relative to the unclustered, singleton members for each deletion mutants relative to wild type gene expression. Hierarchal, k-means clustering organized the families into grouping based on their behavior relative to each remodeling mutant. (Abbreviations: cofac.metpro = cofactor metabolic process, AA.tx = Amino acid transport, Trans.init = translational initiation, CPB = cytoskeletal protein binding, Telo.org = telomere organization, glyco.hydro = hydrolase activity, acting on glycosyl bonds, MT = methyltransferase activity, DNA.met.reg = regulation of DNA metabolic process, Str.Ribo = structural constituent of the ribosome, Nucleus.tx = nuclear transport, Vitamin = vitamin metabolic process, lipid.tx = lipid transport, phos.act = phosphatase activity, PtA = peptidase activity, Regulation.cc = regulation of cell cycle, Transmemb = transmembrane transport, and MC.acid = monocarboxylic acid metabolic process). [file Image1.tif]
